# Grand challenge: addressing the global challenge of healthy aging

**DOI:** 10.3389/fpubh.2025.1657102

**Published:** 2025-07-22

**Authors:** Colette Browning, Marcia Ory, SangNam Ahn, Shane Thomas

**Affiliations:** ^1^Health Innovation and Transformation Centre, Federation University Australia, Ballarat, VIC, Australia; ^2^Institute of Health and Wellbeing, Federation University Australia, Ballarat, VIC, Australia; ^3^Center for Community Health and Aging, Texas A&M University, College Station, TX, United States; ^4^School of Public Health Department of Environmental and Occupational Health, Texas A&M University, College Station, TX, United States; ^5^College for Public Health and Social Justice, Saint Louis University, St. Louis, MO, United States

**Keywords:** aging, public health, multimorbidity, ageism, implementation science, methodology, intergenerational relations

## Abstract

This article reflects upon contemporary issues for the study of healthy ageing and highlights several grand challenges for public health and ageing. Prepared by the Specialty Chief Editors, this Grand Challenge article calls for innovative, equity-focused research and practice to support healthy aging globally. Key research topics include reframing aging as an opportunity, addressing multimorbidity, emphasizing prevention across the life course, applying implementation science, incorporating economic evaluation, using up-to-date data and advanced analytic methods, and promoting inclusion of underrepresented populations.

## Introduction

Frontiers in Aging and Public Health was launched as a section of Frontiers in Public Health in November 2019. Since then, it has published more than 2,000 articles including over 100 Research Topics which provide in-depth insights from coordinated articles highlighting major aging themes. Examples include public health interventions to promote healthy aging, the chronic conditions of aging, mental health and wellbeing, workforce and care issues for aging populations, social and environmental factors in aging and implementation research to accelerate aging and public health research and practice, to name a few. The section adheres to the World Health Organization (WHO) definition of public health as “the art and science of preventing disease, prolonging life, and promoting health through organized efforts of society.” The section was established to challenge the prevailing narrative that frames aging populations as a “burden,” and instead promote healthy aging through public health principles and preventive approaches that enhance health, quality of life, and address social determinants of health. In this grand challenge paper on aging and public health, we argue that our future focus should be on innovative and high-quality research that addresses contemporary and future issues associated with a life course perspective for healthy aging.

## Contemporary issues for the study of healthy aging

While there are many worthy topics, we will just highlight a few deserving further attention.

### Reframing aging

The meaning of aging is not static but changes over time and space. With increases in healthy longevity (1) seventy is often referred to as the new 50, or 80 as the new 60, and even 100 as the new 80. Yet, there is growing recognition that the internalization of current negative stereotypes about aging are health hazards leading to a 7.5-year mortality differential (2). The WHO is leading the charge in combatting ageism globally (3). This involves changing institutional, interpersonal, and intrapersonal views of aging, and developing new systems of Age-Friendly Health Systems better tailored to the needs of older adults and their caregivers (4, 5). Reframing aging also involves caveats about the subtle and not so subtle consequences of employing ageist language, spurring the widespread dissemination of guidelines that provide the dos and don'ts to achieve age-inclusive communication (6). To help address ageism that can stereotype both younger and older populations, aging experts are promoting the benefits of intergenerational relations and living arrangements for all generations (7, 8).

### The challenges of multimorbidity

A feature of aging is its association with chronic conditions and multimorbidity (9), and the social drivers of these health outcomes (10, 11). It is well established that most older people experience multiple diseases or conditions contemporaneously and the global burden of increasing chronic disease is closely related to the global trend of an aging population (12). Research from the U.S. National Council of Aging shows that 95% of US adults aged 60 and older have at least one chronic condition and 78.7% have two or more (13). Australian data show similar trends. As the population ages (14), the rates of chronic diseases and multimorbidity increase (15). Therefore, focusing on a single condition without adequate accounting for comorbidities offers a limited and misleading perspective in healthy aging research. Understanding of comorbidities both physical and mental provides a clearer picture of the challenges facing aging populations and the community resources and clinical services needed to address them (16).

### Consideration of the role of prevention at every life stage

Much of the current and projected global chronic disease burden is provided by preventable diseases that may be mitigated or avoided altogether through modifiable lifestyle and socio-environmental risk factors. The underpinning risk factors for a wide range of chronic diseases are well documented. Many of these chronic diseases share common and co-occurring risk factors such as smoking, poor nutrition (including both malnutrition and obesity), sedentary behavior, excessive alcohol use, high blood pressure, and abnormal blood lipids (17). Social disadvantage, unhealthy environments, and limited access to health care services largely drive these risk factors. Unfortunately, many national health systems make only modest investments in disease prevention, missing critical opportunities to prevent and mitigate these disorders. The response to increased aging related disease burden has been characterized by a focus on ex post facto treatment rather than prevention—akin to placing an “ambulance at the bottom of the cliff.” However, it is important to take a life-course perspective on prevention efforts—such efforts should start early, but it is never too late to better manage conditions and prevent the progression of chronic illnesses and disabilities (1, 18).

The WHO, United Nations, and key government agencies worldwide have invested significantly in studying, preventing, and mitigating non-communicable (chronic) diseases (NCD) and promoting healthy aging. Major initiatives in this area include:

- The UN Decade of HealthyAgeing (2021 to 2030) (19).
- The United Nations Healthy Aging Collaborative (20).
- WHO Age Friendly Cities and Communities (21).
- The European Innovation Partnership on Healthy Aging (22).
- Australian Government Positive Aging is Aging Well (23).
- National Health Committee of China. The 14th Five Year Plan for Healthy Aging (2021–2025) (24).
- The Establishment of the US National Institute on Aging Healthy Aging Program (25).

Many regional and national initiatives echo these global efforts, developing prevention, healthy aging, and NCD programs, often guided (26) by detailed policy input from agencies such as the World Bank (27). The future of some national prevention programs may be influenced by financial downturns and policy changes under many conservative-leaning governments.

However, much research on aging focuses on the impact of individual disorders and fails to recognize the specific needs of older people at risk of multiple chronic conditions and how these conditions may be avoided and mitigated. A key part of our challenge is to improve the quality and impact of aging research for public health.

### Consideration of age, period and cohort effects

Aging is not just about “older people,” or an arbitrary age cut off. Life course perspectives on aging (28) embed the importance of the influence of biological, social and historical influences across the life stages. A surprising aspect of many published aging studies is the lack of basic formal analysis of age (chronological or biological age), period (impact of time, period or history effects regardless of age) and cohort (the impact of shared experiences due to time of birth) effects. Understanding these effects can meaningfully inform the design and implementation of preventive interventions and programs.

### The use of contemporary datasets in aging and public health research

There is increasingly strong evidence that the recent COVID-19 pandemic has significantly altered the landscape of human epidemiology (29). However, most aging studies are silent about the impact of humanity's most recent and significant epidemic, as well as the rapid proliferation of emergent world-wide natural disasters due to climate change. Further many studies use cross sectional analyses of old data that was collected before the onset of this epidemic where it is not possible to even address this issue or other contextual factors such as changing social demographics and healthcare resources (30). It is important that the evidence we use is contemporary and can inform future needs and outcomes (31, 32).

### The use of implementation science in aging research

To improve the uptake of evidence-based research into practice and policy to address healthy aging, we need to consider the application of contemporary implementation science (33).

The first two steps in developing effective interventions and programs are: (1) demonstrating that a particular disorder or cluster of disorders poses a significant burden, and (2) showing that this burden can be reduced through appropriate programs and interventions. The critical third step is to investigate how best to implement these programs. Implementation science plays a pivotal role in addressing this challenge by identifying what is known (the evidence) and how it should be applied in practice to achieve optimal outcomes (34, 35). Conceptual frameworks such as PRISM (36) and RE-AIM (37) offer valuable guidance for such studies. These frameworks stress the importance of context, and the inclusion of broad population groups in intervention efforts and associated research to generalize knowledge and achieve equity (38).

An important feature of contemporary health research and program design is the use of co-design principles (39, 40). The use of such principles enhances consumer participation and engagement in health program design and implementation. The recent emphasis on co-design and consumer engagement arose from critiques of past programs that failed to meet consumer needs and, as a result, compromised their implementation (41, 42). McGill et al. (43) provide a helpful overview of the confused differences between co-production, co-design, co-creation, and co-construction in chronic disease prevention programs. Co-design has been widely applied in the development of health care apps for use by older people (44). Co-design improves uptake of the co-designed tools and processes (and presumably program efficacy) because of the participation in their design, although further work is necessary to verify the validity of this assumption. Many national research grant schemes now have a formal requirement for the inclusion of co-design principles and activities. We encourage the reporting of such work in this section.

### Economic factors in healthy aging research

Economic evaluations of public health programs are also key features of determining the value of interventions and are an important component of implementation science. Unfortunately, such evaluations are not frequently reported (45). Nonetheless, several studies—including umbrella reviews (46, 47)—have assessed the cost effectiveness of public health interventions aimed at promoting healthy aging. Ory et al. (48) revealed how a national chronic disease self-management program in the US could meet the triple aims of health care by improving health, providing better care, and reducing costs. Further Ahn et al. (49) documented specific health care savings through the widespread proliferation of these community-based chronic disease self-management programs and the design of community-friendly tools to track such cost savings. Masters and Anwar estimated the median return on investment (ROI) for public health interventions to be 14.3 to 1. Similarly, Wang and Wang's (50) economic modeling of investment in preventive care in aging societies provides a compelling evidence base for the utility of investing in preventive care for older people. Despite these promising findings, investment in public health interventions remains persistently low in many countries.

Kamal and Hudman (51) analyzed preventive care spending amongst OECD countries and estimated that, on average, preventive care accounted for 2.4% of total health expenditures (51). Despite an overall low percentage of expenditure there were stark differences in expenditures between countries, with Canadian expenditure at 5.9% compared with much lower rates for the U.S. (2.9%), Japan (2.0%) Australia (1.8%), France (1.2%) and Switzerland (1.1%). Data from the WHO Global Health Expenditure Database reflect similar trends, indicating a global pattern of prioritizing curative over preventive health care (52). Paradoxically, an analysis by Schneider et al. (53) of long-term trends in 195 countries over an 18-year period concluded that as country GDPs increase, spending on preventive care tends to decline proportionately.

## Our conclusions on the grand challenges facing public health and aging research

We suggest addressing the following challenges in research in public health and aging. We propose:

- Reframe global aging from a problem to an opportunity to create new social structures and systems of care.
- Focus on implementation science to better understand how to translate evidence into effective programs.
- Consider both physical and mental comorbidities in the study of disorders to more accurately reflect the true burden of disease among older adults.
- Emphasize studying the costs of disorders and the benefits of their prevention and treatment to strengthen the evidence base for health policy.
- Employ advanced analytic methods—such as accounting for age-period-cohort effects, conducting risk analyses, and applying outcome prediction modeling -to enhance the rigor and quality of published research.
- Ensure that secondary analyses use up-to-date data to maintain relevance and accuracy in findings.
- Include underrepresented populations, including those from low socioeconomic or rural backgrounds, displaced persons such as migrants and refugees, and individuals with mental health conditions to generalize research and practice implications.
